# Women Have Reduced Ability to Discriminate Body Odors During the Withdrawal Period of Oral Contraception

**DOI:** 10.1007/s12078-019-09273-9

**Published:** 2019-12-06

**Authors:** Yaara Endevelt–Shapira, Liron Pinchover, Ofer Perl, Ella Bar, Ayelet Avin, Noam Sobel

**Affiliations:** Department of Neurobiology, Weizmann Institute of Science, 76100 Rehovot, Israel

**Keywords:** Olfaction, menstrual cycle, body odor, oral contraception

## Abstract

**Introduction:**

Women’s olfactory perception varies across the menstrual cycle. The influence of oral contraceptives on this variability remains unclear.

**Methods:**

To further estimate this, we assessed discrimination performance for both body odors and ordinary odorants in 36 women, 18 naturally ovulating, and 18 using oral contraceptives. Each participant was tested once a week over the course of a month, and data was then parsed into menstrual phases.

**Results:**

In naturally ovulating women, at the transition from follicular to luteal phases, there was a decline of 19% (*p* = 0.003) in olfactory discrimination of body odors but not ordinary odorants. In turn, in women using oral contraceptives, only at a later time of the month, at a point corresponding to the late luteal phase and shift from post-ovulation to pre-menstruation, was there a decline of 20% (*p* = 0.002) in olfactory discrimination performance. Moreover, when we reorganized the data from women using oral contraceptives in order to separately assess the contraceptive withdrawal period (the few days off pills), we observed a 23% reduction (*p* = 0.01) in discrimination accuracy of body odors but not ordinary odorants during this time alone.

**Conclusions:**

Women have reduced ability to discriminate body odors during the withdrawal period of oral contraception.

**Implications:**

If women indeed consider men’s body odor in their mate selections, then the oral contraception withdrawal period may not be the best time to make such decisions.

## Introduction

Olfactory sensitivity and perception in women vary across the menstrual cycle (Doty and Cameron 2009a). Most reports imply high sensitivity during the follicular pre-ovulation and ovulation phases compared with the luteal and menstruation phases (Navarrete-Palacios et al. 2003; Vierling and Rock 1967). Further indications suggest this link between menstrual phase and perception is odor-type dependent (Doty and Cameron 2009a; Lundstrom et al. 2006; Mair et al. 1978). Although this variability is likely linked to cycling hormones (Caruso et al. 2001; Renfro and Hoffmann 2013), the specifics of the mechanism underlying this variation remain unclear. In women using oral contraceptives (OC), the natural hormonal cycle is pharmacologically altered, and this could potentially provide insight into the role of cycling hormones in olfactory perception. To date, observations regarding the impact of OCs on olfactory perception are mixed, ranging from reports of OC-related long-term improvement in olfactory performance (Derntl et al. 2013), through reports of unchanged olfaction with OCs compared with naturally ovulating women (Derntl et al. 2013; Doty et al. 1981), and on to reports of OC-related deterioration in olfactory performance compared to follicular and pre-ovular phases of naturally ovulating women (Caruso et al. 2001).

The potential impact of OCs on a subset of olfaction, namely, perception of social chemosignals and body odors, has received special attention. Moreover, here, the results have been less variable: Women’s preferences for men’s body odors vary across the menstrual cycle: as fertility peaks, olfactory preference increases for odors of men who are perceived as dominant (Havlicek et al. 2005), symmetrical (Gangestad and Thornhill 1998; Rikowski and Grammer 1999), and with dissimilar MHC (Wedekind et al. 1995). No such preferences were observed in non-fertility phases of the cycle or in women using OCs (Gangestad and Thornhill 1998; Roberts et al. 2008; Wedekind et al. 1995). Combined with findings of general OC-related reductions in sensitivity to social chemosignals (Lundstrom et al. 2006; Renfro and Hoffmann 2013), these findings generated heightened public interest because they raised the possibility that women who formed and solidified a relationship while using OCs were not privy to potentially meaningful olfactory information such as MHC fit. We set out to ask whether olfactory performance in tests involving body odors and mixtures of ordinary chemical odorants is altered along the menstrual cycle. We tested this in two cohorts: naturally ovulating women and women who use OCs. Since some of the variability across the menstrual cycle that was observed in previous studies may reflect the inevitable variance in across-subject designs (different women at each menstrual phase), in the current study, we applied a within-subject design paradigm in which over the course of a month, we weekly assessed olfactory performance in the same women, one group naturally ovulating and another group using OCs.

## Materials and Methods

### Location

All experiments were conducted at the Weizmann Institute of Science in rooms specifically designed for human olfaction studies. These rooms are coated in stainless steel and subserved by HEPA and carbon filtration in order to prevent cross-contamination across experiments and conditions.

### Participants

A total of 36 participants took part in the reported experiments after providing written informed consent to procedures approved by the Weizmann Institute IRB committee.

The participants were assigned into two different groups: women who were using oral contraceptives (OCs, *n* = 18, Age = 24.8 ± 1.6 years) and women who were not using oral contraceptives for at least 10 months before the experiment (NOC, *n* = 18, age = 24.7 ± 1.6 years). All participants reported their last menstruation date and their typical cycle duration. Cycle length in the naturally ovulating women was in the range of 26 to 35 days (mean = 28.8, std = 2.5) (Table 1). The participants in the OC group reported their birth control pill type and brand. All were combined monophasic pills, based on estrogen and progestogen derivatives. All participants had self-proclaimed normal olfaction, and olfaction was further estimated to the extent that they here participated in a repetitive olfactory discrimination task. All participants but one performed significantly above chance (mean performance score = 0.56 ± 0.09, one sample two-tailed *t* test with 0.33 as the hypothetical mean, t(35) = 15.3, *p* < 0.0001). The one participant who was at chance was excluded from further analysis. Five participants were smokers (two in the OC group, and three in the NOC group). We later compared discrimination performance in these smokers versus the non-smokers and observed no differences (Z = 0.33, *p* = 0.74). Thus, although smoking is a factor in olfaction (Katotomichelakis et al. 2007), it likely did not impact our results.

**Table 1:** Cycle menstrual day in each of the experimental session. * represents the participants that were included in the WD/CP analysis

Subject	First session	Second session	Third session	Fourth session	Cycle duration
NOC1	20	27	6	13	28
NOC2	26	5	12	19	28
NOC3	11	18	25	4	28
NOC4	18	25	3	9	28
NOC5	22	4	10	17	26
NOC6	14	21	28	7	32
NOC7	17	24	3	10	28
NOC8	25	4	11	18	28
NOC9	13	20	27	2	32
NOC10	9	16	23	4	26
NOC11	23	2	9	16	28
NOC12	12	21	28	2	33
NOC13	19	28	35	7	35
NOC14	16	25	4	11	28
NOC15	12	18	25	5	28
NOC16	18	23	3	10	27
NOC17	28	6	13	21	27
NOC18	5	11	18	26	28
OC1*	6	13	20	27	
OC2*	2	9	16	23	
OC3	25	4	11	18	
OC4*	24	3	10	17	
OC5	18	25	4	11	
OC6	12	19	26	5	
OC7*	24	3	11	17	
OC8*	14	21	28	7	
OC9*	7	14	21	28	
OC10*	8	15	22	1	
OC11	25	4	11	18	
OC12*	27	6	13	20	
OC13*	14	21	28	7	
OC14*	23	2	9	16	
OC15*	7	14	21	28	
OC16	4	11	18	25	
OC17*	4	11	18	1	
OC18*	7	14	20	27	

### Olfactory Tasks

#### Body Odor Discrimination Task

##### Body Odor Collection

Fifteen donors (7F, range 23–46 mean age 30.5 ± 6.1) were provided with brand new 100% cotton white T-shirts. The donors were instructed to wear the shirts for two consecutive nights. The donors were further instructed to avoid consuming food ingredients that alter body odor (fenugreek, asparagus, curry, etc.) for at least 2 days prior to body odor sampling. Additionally, during sampling days, the donors were asked not to use soap, shampoo, conditioner, or deodorant. When not worn, T-shirts were kept inside closed glass jars stored in the donors’ home freezer. When obtained from donors and brought back to the lab, jars were stored at − 20 °C to prevent bacterial growth.

##### Shirt Sniffing Device

**Fig. 1.**
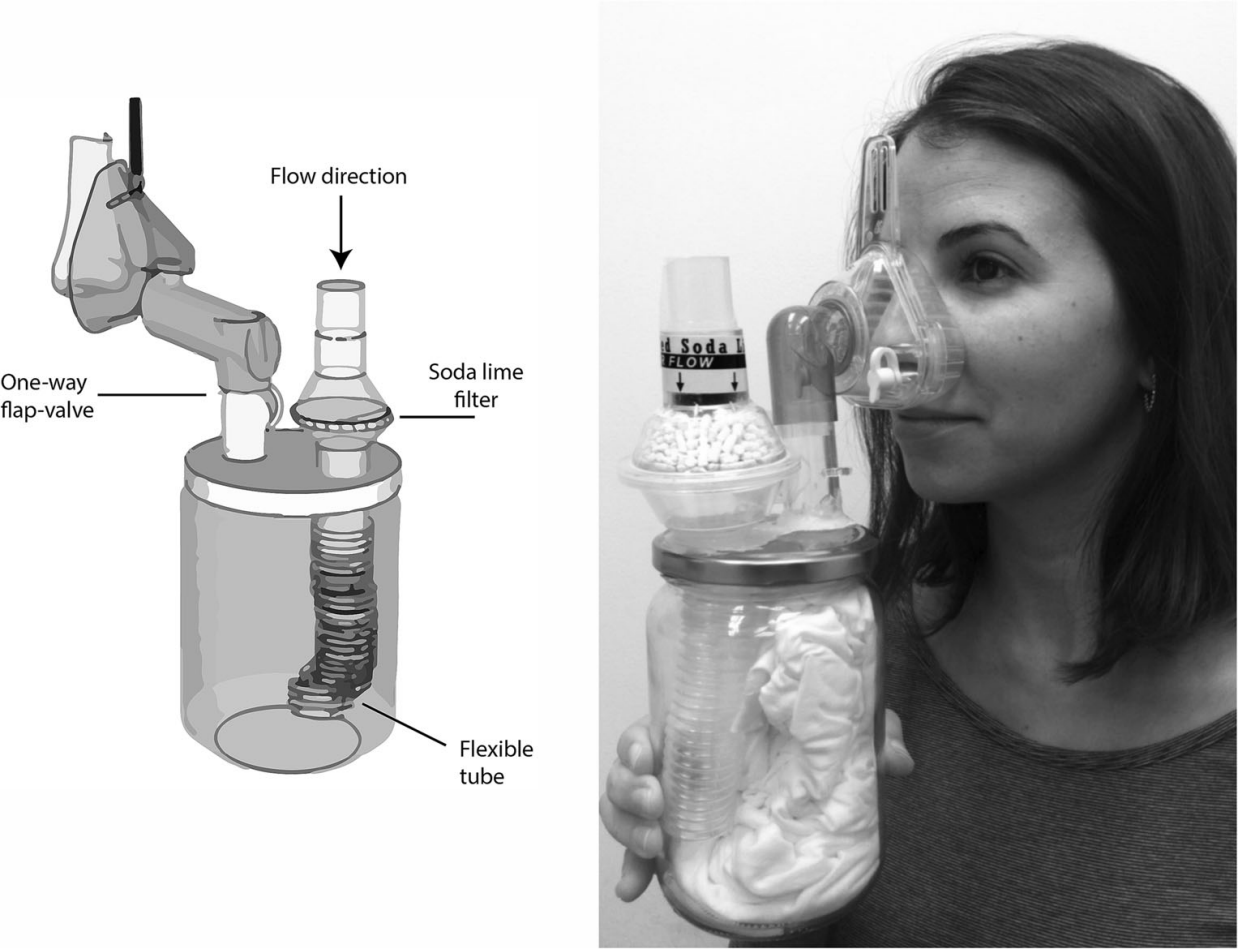
The shirt-sniffing device (SSD). To standardize body-odor sampling we developed the SSD. This consisted of a glass jar containing the T-shirt, with an air intake port via soda lime filter, and air sampling port via one-way flap valve into individual-use airtight nose mask. This arrangement assured that environmental odors did not contaminate the sample during the sampling process. The recognizable person in the figure is a co-author and not a participant

On the morning of the first day of the experiment, shirts were thawed inside the jars to avoid condensation or humidity. Next, they were cut by a sterile pair of scissors into two longitudinal pieces, such that in each half contained one axillary area. Each half was then placed inside a shirt sniffing device (SSD)—a glass jar, covered by a cap fitted with an air filter, an inhalation mask, and a one-way flap-valve. The SSD prevented contamination of the body odors by odorants emitted from the participants, or from the environment (Fig. 1). The shirts were replaced by new ones from the same donors after two sessions to avoid odor attenuation and hence participants’ ability to judge by odor intensity rather than by genuine discrimination. Participants performed a three-alternative forced-choice task between SSDs. In each trial of the discrimination task, two of the SSDs contained a shirt that originated from the same donor and the third contained a shirt from a different donor. The SSDs were presented to the subjects by a cross gender experimenter, and trials were not time-limited and randomly ordered. Notably, each session consisted of three trios of a male donor with a male distracter, three trios of a female donor with a female distracter, three trios of a male donor and a female distracter, and three trios of a female donor with a male distracter. Each of the trios comprised a different combination of donors and was presented only once throughout a session.

##### Chemical Mixtures Discrimination Task

We used a three-alternative forced-choice discrimination task, this time between mixtures of ordinary chemical odorants. Participants engaged in 12 trials; in each trial, three opaque jars were presented to the participant in a randomized order. Two jars contained an identical odorants mixture and the third contained a different mixture (all equated for perceived intensity). Participants were allowed a single 2-s long sniff at each odor presentation and were then instructed to select the jar that contained the odd odor. Sniff duration was imposed by matching inhalation to a concurrent auditory tone. This limitation to a single sample/sniff per stimulus was introduced because overexposure to the chemical mixtures can be overwhelming, leading to habituation and reduced performance later on in the task. Two types of odorants mixture trios were used. Each trio is composed of two different mixtures, generated using 5 or 6 components, with a single odorant replaced in each “dissimilar” mixture in each trio (see Table 2 for complete list of mixtures used).

**Table 2:** Mixture discrimination task

Odor number	Odorant	CAS number	Dilution in μL/10 mL mineral oil	Mix1	Mix2	Mix3	Mix4
1	Isoamyl acetate	123-92-2	152	+	+		
2	Isopropylbenzene	98-82-8	166			+	+
3	1-pentanol	71-41-0	124	+	+	+	+
4	1,7-octadiene	3710-30-3	124	+	+		+
5	2-heptanoe	110-43-0	197	+		+	
6	Heptanal	111-71-1	216		+	+	+
7	3-methyl-2-buten-1-ol	556-82-1	110	+	+	+	+
8	Propyl butyrate	105-66-8	128	+	+		

### Procedures

Each participant was tested once a week over the course of a month. The participants came to lab on specific dates that were pre-scheduled regardless of cycle phase, providing for randomization of cycle phase across participants (Table 1). In each session, the participants conducted two olfactory tasks—olfactory discrimination of body odors and olfactory discrimination of ordinary chemical mixtures. The tasks were in blocks for each type of odorant. We used two identical rooms—in one room, the participants conducted the mixture discrimination task and in the other, they conducted the body odor task. The order of the tasks was counterbalanced across participants. For each participant, we calculated the fraction (0-1) of correct discriminations for each task and session.

### Statistical Analysis

To estimate sample size, we conducted a power analysis using G*Power software (Faul et al. 2007) applied to data by Renfro. et al (Renfro and Hoffmann 2013), which suggested at least 16 participants per group in a between-participants analysis to achieve power of 0.6 at alpha = 0.05. Prior to analysis, we estimated data distribution. The mean olfactory scores were normally distributed (*P* > 0.05, Shapiro–Wilk normality test), and the variances homogeneous among groups (*P* > 0.05 Levene’s homogeneity of variance test). Next, an analysis of variance (ANOVA) was applied to the data, followed by planned two-tailed *t* tests, Tukey HSD corrected for multiple comparisons where appropriate.

### Data Sorting by Menstrual Cycle Phases

We first classified experimental sessions in the NOC group into four menstrual phases: menses, proliferative (pre-ovulation and ovulation), post ovulation, and pre-menstrual. The proliferative period is the one variable in length and therefore contributes to variations in overall cycle length (Fehring et al. 2006). In our ordering of the data, only the proliferative period of the cycle was variable; hence, participants with longer cycle had a longer proliferative period and vice versa. Data of the OC group were artificially ordered in the same manner based on the day of menstruation.

### Data Sorting According to Withdrawal Days

We reassigned OC data into withdrawal days (WDs) and contraceptive phase (CP). Participants used contraceptives under two regimens; a regimen of 21 days followed by seven withdrawal days, and a regimen of 24 days followed by four withdrawal days. Withdrawal days were strictly defined as the last 2 days of the cycle followed by the first 3 days of the cycle for participants using the 21 + 7 regimen, or the last day followed by the first day of the cycle for the 24 + 4 regimen. The remaining days were considered as the contraceptive period. Thus, the CP is comprised of three experimental sessions while WDs are comprised of one experimental session. Participants who did not have olfactory scores in the defined withdrawal days (because these did not fall on our randomly timed weekly testing) were not available for this particular analysis, retaining 13 participants in the OC group for this analysis alone.

## Results

### Changes in Olfactory Discrimination Performance Across the Menstrual Cycle

To test whether olfactory performance changes across the menstrual cycle, and whether this differs in OC and NOC as a function of odor type, we conducted a repeated-measures ANOVA on olfactory discrimination scores with conditions of menstrual cycle phase (menses/proliferative /post ovulation/pre-menses) and odor type (BO/Mix) and an independent variable of group (OC/NOC). We observed main effects of odor type (F(1,33) = 14.3, *p* < 0.001) and phase (F(3, 99) = 5.2, *p* = 0.002), yet no main effects of group (F(1,33) = 0.008, *p* = 0.93). The main effect of odor type reflected that overall performance in the chemical mixtures discrimination task was lower than the performance in the body odor discrimination task (Mix = 0.53 ± 0.12, BO = 0.61 ± 0.10, t(34) = 3.8, *p* = 0.0005, Cohen’s d = 0.77). The effect of phase reflected that overall olfactory discrimination performance was lower in the last phase (pre-menses = 0.51 ± 0.14) compared with menses and proliferative phases (menses = 0.59 ± 0.13, proliferative = 0.60 ± 0.14, both t(34) > 3.1, both corrected Tukey HSD *p* < 0.01, all Cohen’s d > 0.64). The lack of group effect reflected nearly equal performance in NOC and OC (NOC = 0.57 ± 0.10, OC = 0.57 ± 0.08). We further observed no interactions with odor type (all F(3,99) < 1.2, all *p* > 0.3), yet a significant interaction of phase × group (F(3, 99) = 3.6, *p* = 0.017). In addition, to verify that the effects we observed were not a reflection of learning, we analyzed discrimination performance across sessions, regardless of menstrual phase. A repeated-measures ANOVA with conditions of session and odor and an independent factor of group revealed no main effect of session (F(3,99) = 1.6, *p* = 0.20). This bodes against a significant contribution of learning to performance over the progression of this study. To further explore the aforementioned significant interaction of phase × group (F(3, 99) = 3.6, *p* = 0.017), we next conducted post hoc analyses on each group separately.

### Naturally Ovulating Women: Reduced Discrimination of Body Odors in the Luteal Phase

In the NOC group we observed significant main effects of odor type (Mix = 0.53 ± 0.12, BO = 0. 61 ± 0.11, F(1,16) = 9.0, *p* = 0.009) and phase (F(3, 48) = 3.0, *p* = 0.038), indicating lower olfactory performance in post ovulation phase compared with proliferative phase (proliferative = 0.62 ± 0.16, post ovulation = 0.52 ± 0.09, t(16) = 2.8, corrected with Tukey HSD , *p* = 0.05, Cohen’s d = 0.78). Yet, the ANOVA revealed no interaction of phase × odor (F(3,48) = 1.2, *p* = 0.33).

**Fig. 2:**
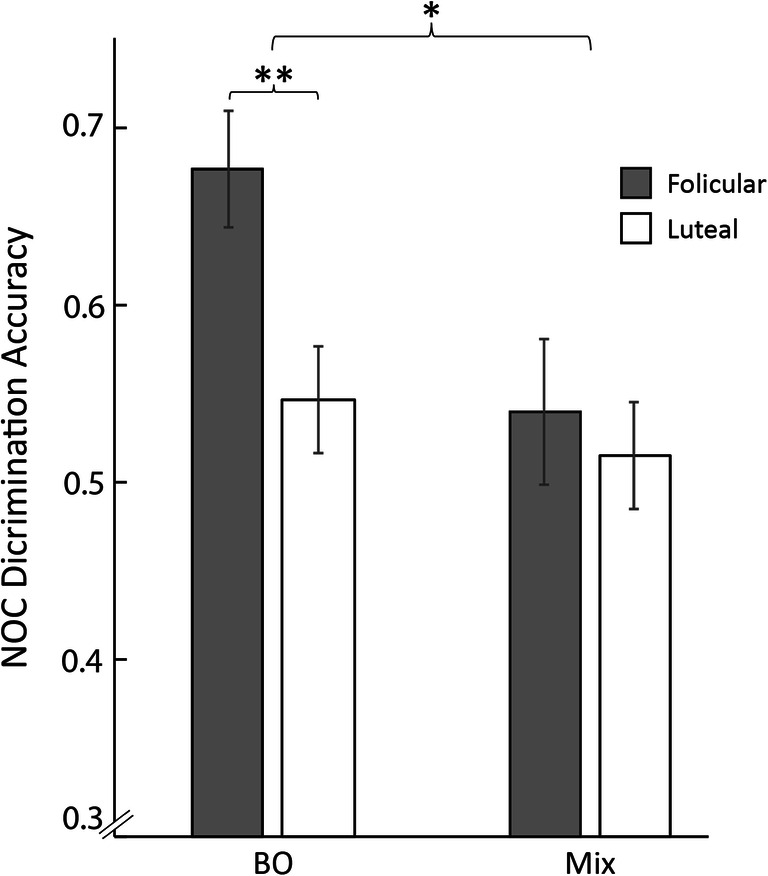
Discrimination accuracy for body odors drops during the luteal phase in naturally ovulating women. Mean ± SEM fraction (0–1) of accurate discrimination in the follicular (gray bars) and luteal (white bars) phases, both for body odors (BO) and chemical mixtures (MIX), in naturally ovulating women (not using oral contraceptives (NOC). *p < 0.05; ***p < 0.001

In addition, following the observed difference in olfactory performance between the proliferative and the post-ovulation phases, we continued to compare olfactory performance between the follicular (mean scores of menses and proliferative) and luteal (mean scores of post ovulation and pre-menses) phases. We conducted a repeated-measures ANOVA with conditions of phase (follicular/luteal) and odor type (BO/Mix). The ANOVA revealed a main effect of phase (follicular = 0.61 ± 0.13, luteal = 0.53 ± 0.10, F(1,16) = 9.7, *p* = 0.007), a main effect of odor type (F(1,16) = 9.0, *p* = 0.009), and a significant interaction of phase × odor type (F(1,16) = 5.9, *p* = 0.027). The significant interaction reflected significantly reduced performance in the BO task (follicular = 0.68 ± 0.13, luteal = 0.55 ± 0.12, t(16) = 4.6, *p* = 0.0002, corrected Tukey HSD, *p* = 0.003, Cohen’s d = 1.04), but no difference between phases in the mixture discrimination task (follicular = 0.54 ± 0.16, luteal = 0.51 ± 0.12, t(16) = 0.69, *p* = 0.52) (Fig. 2). In other words, consistent with previous reports of odor type-dependent shifts in olfactory performance across the menstrual cycle (Doty and Cameron 2009a; Lundstrom et al. 2006; Mair et al. 1978), we observed significantly higher olfactory performance pre-ovulation for one type of stimulus (body odors) but not for the other type (ordinary odors).

### Women Using Oral Contraceptives: Reduced Discrimination of Body Odors in the Contraceptive Withdrawal Period

**Fig. 3:**
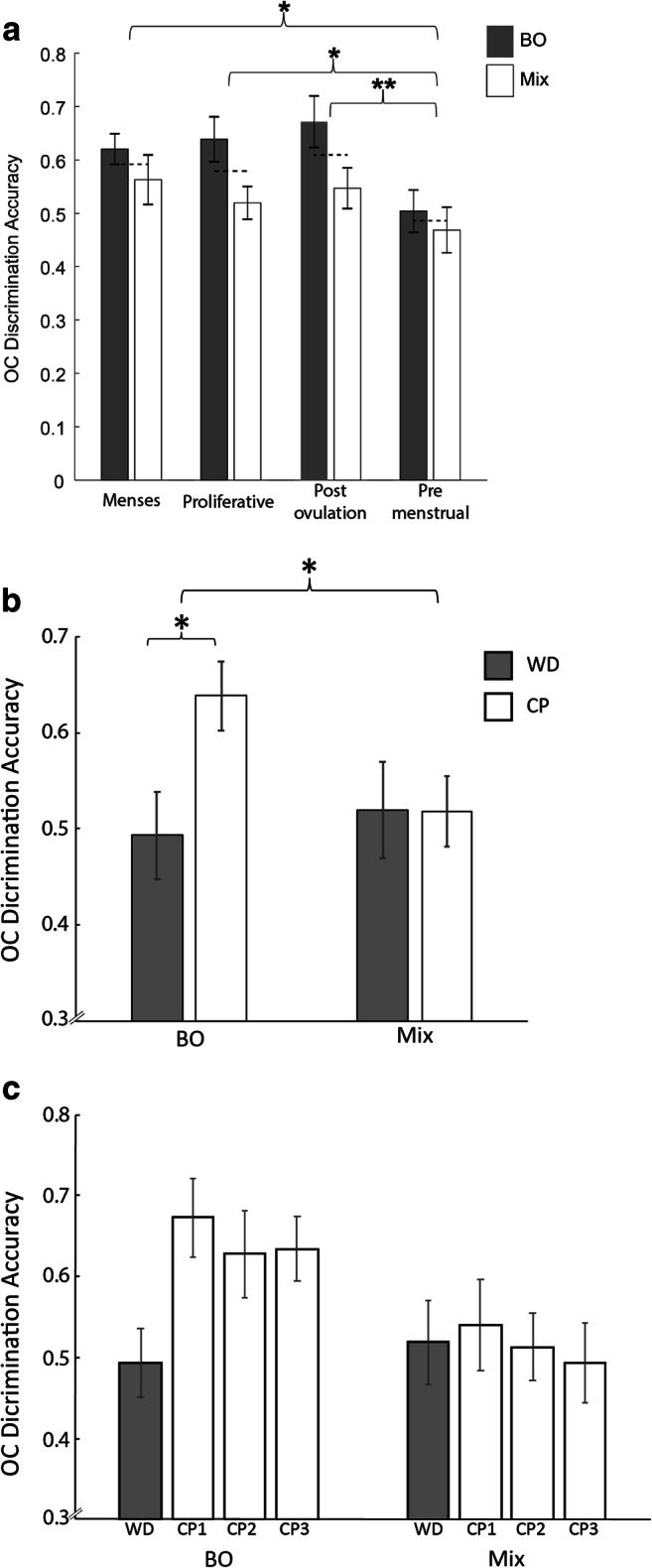
Discrimination accuracy for body odor drops during the withdrawal period from oral contraceptives Mean ± SEM fraction (0–1) of accurate discrimination in women using oral contraceptives (OC), **a** across phases of the menstrual cycle (1–4) for both body odor (BO) (gray bars) and chemical mixtures (MIX) (white bars). The horizontal dashed line represents the average score of the tasks per phase and (**b**) comparing withdrawal period (WD, gray) with the average contraceptive period (CP, white) of the two olfactory tasks. **c** Performance at each of the three CPs separately (white bars) and WD (gray). *p < 0.05; **p < 0.01

In the OC group, the ANOVA revealed a main effect of odor type (mix = 0.52 ± 0.12, BO = 0. 61 ± 0.10, F(1,17) = 6.1, *p* = 0.024), no interaction of phase with odor type (F(3,51) = 0.77, *p* = 0.52), yet a significant main effect of phase (F(3,51) = 6.2, *p* = 0.001), indicating that olfactory performance was significantly lower during the last phase (pre-menses = 0.49 ± 0.12) compared with all other phases (menses = 0.59 ± 0.10, t(17) = 2.9, *p* = 0.01 , Cohen’s d = 0.94, proliferative = 0.58 ± 0.11, t(17) = 2.78, *p* = 0.013, Cohen’s d = 0.78, post ovulation = 0.61 ± 0.12, t(17) = 3.7, *p* = 0.002, Cohen’s d = 0.99, all Tukey HSD corrected, *p* < 0.05) (Fig. 3a). In other words, we observed a sharp decrease in olfactory performance during the days before menstruation in the OC group. Given that women using OCs enter a withdrawal period at the end of each cycle, we speculated that the observed decrease in olfactory performance is related to this withdrawal period. With this in mind, we went further to ask whether olfactory perception is altered specifically during the withdrawal phase in the OC group. To this end, we re-arranged the data according to withdrawal days (WDs) and contraceptive days (CPs).

To ask whether olfactory performance differed during the withdrawal days of the oral contraceptive group, we conducted an ANOVA with conditions of odor type (body odors/chemical mixtures) and time (CP/WD). The ANOVA revealed a significant interaction of odor type × time (F(1,12) = 7.5, *p* = 0.018), reflecting that during the withdrawal days, there was significantly lower olfactory performance in the body odor task (CP = 0.64 ± 0.12, WD = 0.49 ± 0.16, t(12) = 3.6, *p* = 0.004, corrected Tukey HSD = 0.01, Cohen’s d = 1.06), but not to ordinary chemical mixtures (CP = 0.52 ± 0.13, WD = 0.52 ± 0.17, t(12) = 0.03, *p* = 0.98) (Fig. 3b). This result survives Bonferroni correction for the added multiple comparisons. We note that two participants were on a different temporal OC regimen: 24–4 rather than 21–7 days of use-to-withdrawal. We observe that this effect remained significant without these two participants (t(10) = 3.2, *p* = 0.0097, Cohen’s d = 1.02), and that each of these two participants had decreased performance during the withdrawal as well. To verify that the observed differences in BO were not merely a reflection of variation in CP scores that were averaged, we tested for differences across CP weeks (weeks 1–3 following WD) and observed no main effect of time (F(2,24) = 0.71, *p* = 0.50) and no interaction of time × odor (F(2,24) = 0.086, *p* = 0.92. BO: CP1 = 0.67 ± 0.16, CP2 = 0.63 ± 0.19, CP3 = 0.63 ± 0.15; MIX: CP1 = 0.54 ± 0.19, CP2 = 0.51 ± 0.14, CP3 = 0.49 ± 0.17. Finally, one may ask why do we refer to this as a reduction in performance during WD rather than an increase in performance in CP? To clarify this, we depict performance at each of the three CPs separately (Fig. 3c) and observe that whereas the ordinary odorant mixture performance remains constant across CPs and WD, body odor performance remains constant across the three CPs but drops during WD (Fig. 3c).

Taken together, these results suggest that the differences we observed in sensitivity to BO resulted from a reduction in olfactory performance during the WD phase in the OC group.

## Discussion

We asked whether olfactory performance is altered during the menstrual cycle both in naturally ovulating women (NOC group) and in women consuming oral contraceptives (OC group). We found that overall olfactory performance across tasks was comparable in OC and NOC. This finding is consistent with one report of equal performance in OC and NOC (Derntl et al. 2013), yet inconsistent with another report of OC-related deterioration in olfactory performance compared to follicular and pre-ovular phases in naturally ovulating women (Caruso et al. 2001). This contradiction may reflect the difference in olfactory tasks used: whereas we used discrimination with odorant mixtures, the study that found differences used threshold tests with mono-molecular stimuli. Here, whereas performance was equal cross groups, it varied in each group over time. Naturally ovulating women had higher sensitivity during the follicular phase (prior to ovulation) compared with the luteal phase, specifically for body odors. This finding is consistent with previous reports (Caruso et al. 2001; Doty et al. 1981; Mair et al. 1978; Navarrete-Palacios et al. 2003; Vierling and Rock 1967) and stresses the possible role of olfaction during the reproductive menstrual phase.

Additionally, we observed decreased olfactory sensitivity during the last phase of the cycle in the OC group compared with all other phases. We found that during the withdrawal period, women exhibited significantly reduced discrimination abilities for body odors. We did not, however, observe such withdrawal-associated alterations in sensitivity to ordinary odorant mixtures. Body odors play a significant role in human mate selection, and hormonal state shapes body odor preferences (Gangestad and Thornhill 1998; Havlicek et al. 2005; Rikowski and Grammer 1999). The interplay between this phenomenon and OC use had gained public attention because such studies implied that women using OCs might be making the “wrong” mate selections, olfaction-wise, only to discover this when they discontinue OC use (Roberts et al. 2008). The aforementioned studies measured odor attractiveness ratings, and here, we measured discriminability. Thus, our results add to the notion of an impact of OC on body odor perception, but in a more complex manner. For body odor discrimination (rather than preference as was previously measured), we did not observe a difference between NOC and OC, but rather a significant reduction during OC withdrawal. Such reduction suggests poorer discrimination ability when contrasted with the contraceptive period in the same women.

The mechanistic underpinnings of the observed reduced performance remain unknown.

All participants in the OC group used monophasic contraceptive pills based on estrogen and progesterone derivatives. We speculate that the decrease in olfactory sensitivity to body odors during withdrawal days may be related to a sudden decrease in estrogen and/or progesterone levels associated with the pause in intake. The observed effects could be mediated either directly by the decrease in these exogenous hormones or indirectly through the consequential diminished inhibitory effect on the pituitary–ovarian axis, resulting in increase of luteinizing hormone and follicle-stimulating hormone (FSH) (Baerwald et al. 2004; van Heusden and Fauser 2002; Vandever et al. 2008; Willis et al. 2006). Whereas we do not think that the olfactory system has evolved to adjust for oral contraception (...), from a hormonal perspective, one can loosely relate the oral contraceptive phase with pregnancy, and the withdrawal period of contraception with postpartum. More specifically, elevated estrogen is clearly associated with improved olfaction (Doty and Cameron 2009b), and estrogen levels are indeed elevated in both oral contraception and pregnancy (although the common notion of improved olfaction in pregnancy awaits added verification; Cameron 2014; Ochsenbein-Kölble et al. 2007). In turn, there is a sharp decrease in estrogen postpartum and in the oral contraceptive withdrawal phase, which here was associated with reduced discrimination of body odors.

We would like to acknowledge several limitations of the current study: First, we set out to test for differences between groups as a function of menstrual phase, yet observed differences specifically during withdrawal. This restricted our relevant cohort size for the final analysis, and thus limited the power of our observations. Second, although all participants used monophasic combination pills (using estrogen and progesterone derivatives), the estrogen dosage varied between the different pills that were used by the participants. In the current data, there was no correlation between estrogen dosage and change in performance (Spearman, r = 0.28, *p* = 0.25), but this observation falls short of a systematic titration, and this deserves future attention. Third, the body odor discrimination task included odors obtained from both men and women donors, resulting in different sex combinations. One may ask whether the gender of the body odor donor may explain differences in discrimination performance throughout the menstrual cycle, yet our current study was not designed to address this question as we had only three repetitions per combination. Finally, we cannot rule out that the differential impact of withdrawal on the perception of body odors versus ordinary odorants was a reflection of the difference in the tasks (limitation to one sniff in ordinary odorants) rather than the difference in the nature of the stimuli. This difference in task was a *necessary evil* to prevent habituation and equate performance, but its potential added contributions must be kept in mind.

To conclude, we report a difference in olfactory discrimination of body odors between the withdrawal and contraceptive periods. The implications of our findings in this respect are twofold: First, we caution olfactory psychophysicists to be cognizant of these fluctuations in performance during the withdrawal period, specifically in social chemosignaling research. Second, a more general implication is that if humans indeed make behavioral decisions based on body odor, then the OC withdrawal period may not be the best time to make such decisions.
